# Knowledge and practice of testicular self-examination among secondary students at Ntare School in Mbarara District, South western Uganda

**DOI:** 10.11604/pamj.2019.33.85.15150

**Published:** 2019-06-06

**Authors:** Catherine Atuhaire, Ambrose Byamukama, Rosaline Yumumkah Cumber, Samuel Nambile Cumber

**Affiliations:** 1 Mbarara University of Science and Technology, Faculty of Medicine, Department of Nursing, Mbarara, Uganda; 2 Faculty of Political Science, University of KwaZulu-Natal, Durban, South Africa; 3 Faculty of Health Sciences, University of the Free State, Bloemfontein, South Africa; 4 Section for Epidemiology and Social Medicine, Department of Public Health, Institute of Medicine (EPSO), The Sahlgrenska Academy at University of Gothenburg, Gothenburg, Sweden; 5 School of Health Systems and Public Health Faculty of Health Sciences, University of Pretoria Private Bag X323, Gezina, Pretoria, South Africa

**Keywords:** Knowledge, practice, testicular, self-examination, Uganda

## Abstract

**Introduction:**

Testicular self-examination (TSE) is a screening technique that involves inspection of the appearance and palpation of the testes to detect any changes from the normal. Globally, the incidence of cancer has increased among which is testicular cancer (TC). Data on this topic among male secondary school adolescents in Uganda is limited therefore this study sought to assess the knowledge and practice of testicular self-examination among secondary students at Ntare School, Mbarara District in south western Uganda. The objective of the study is to assess the knowledge and practice of testicular self-examination among secondary students at Ntare School in Mbarara district, south western Uganda.

**Methods:**

We conducted a descriptive cross-sectional quantitative study among 165 students. Recruitment was made using simple random sampling technique. Respondents were selected among advanced level (A’ level) male students studying at Ntare School in Mbarara district, south western Uganda. Structured self-administered questionnaires were used for data collection.

**Results:**

Of the male students, 41.8% reported to have knowledge about TSE and only 23.6% practiced TSE. Most students rated their knowledge of TSE to be below 5 (from 1-10). Of the 39 students who admitted performing TSE, only 16 did so as recommended (monthly).

**Conclusion:**

The knowledge and practice of TSE were low among adolescent secondary school boys in Ntare School in Mbarara District, south western Uganda. This suggests that these students are unaware of the value of this personal health promotion tool which is fundamental in early diagnosis of testicular cancer.

## Introduction

Testicular self-examination (TSE) is an easy screening technique that involves inspection and palpation of the testes to detect any changes and for early detection of testicular cancer [1]. In this procedure, males check their own testicles in order to rule out any unusual lumps or bumps, which maybe the first sign of testicular cancer [2]. It is recommended for the early detection of testicular cancer in males [3]. Having knowledge is highly associated with TSE practice and is also important in helping males know the importance of TSE as this will help to prevent late-stage diagnosis of testicular cancer [1]. The TSE procedure is essential as an effective means to promote testicular health, self-awareness and wellness among males [4]. Testicular cancer is a disease in which cells become malignant (cancerous) in one or both testicles [5]. It is the most common cancer among 15 to 34 year-old males [4]. The most common symptom is a painless swelling in the early period [6]. Recent data suggest that rates for new testicular cancer cases have been rising on average of nearly 1% each year in the past decade [4]. Mistry *et al.* [7] estimated the lifetime risk of developing testicular cancer to be 1 in 210 (for men in the UK), while incidence rates have been shown to peak at around 17 per 100,000 in the 25 to 34 age category. Testicular cancer incidence is strongly related to age, with the highest incidence rates overall being in younger males.

Siegel *et al.* [8] recommend a testicular self-examination as part of a routine cancer-related checkup, designed to facilitate early detection of testicular cancer and it should be performed monthly by males beginning at age of 15 years. Besides testicular cancer assessment, males with knowledge about TSE can easily check themselves for male-specific urogenital health concerns, including scrotal abnormalities like varicoceles, hydroceles, unusual lumps, testicular swellings which are all risk factors of testicular cancer [9]. Every male at puberty should be taught and encouraged to perform a monthly TSE for the purpose of detecting testicular tumors or other scrotal abnormalities [10]. It is reported that a better prognosis with 5-year survival rate is likely in males who effectively carryout TSE and identify a lump in its early stages [11]. Education and instruction for men on the normal shape and texture of the testicles, plus information regarding signs and symptoms associated with TC could be a critical component in reducing treatment delay [12, 13]. Brenner *et al.* [14] state that screening for testicular cancer by a health care provider and/or testicular self-examination (TSE) is rarely performed, or underperformed, with little or no documentation regularly. There is an increasing incidence of cancer among which is testicular cancer (TC). Globally, incidence rates of TC are rising among the 15 to 54 year old males, with the majority of those cases affecting males under the age of 40 years [15]. TC accounts for 1 to 2% of all cancers occurring in males, with approximately 2,000 new cases diagnosed in the UK each year [7]. In the United States, about 8,000 men are diagnosed with testicular cancer and about 390 men die of testicular cancer each year [5].

In USA, ideally males beginning at the age of 15 years are supposed to be provided with the knowledge to practice testicular self-examination by physicians or nurses, as recommended by the American Cancer Society [8]. Cancer registries and support groups in the United Kingdom recommend that young men should be provided with a basic awareness of TC and the knowledge that medical advice should be sought incase testicular lumps or masses are found [16]. Even when TSE is recommended at an early age, a study done in the USA by [3] showed that 46% of respondents reported performing TSE and 51% reported not performing. According to Ugboma *et al.* [17], the awareness of testicular cancer is poor among Nigerian men and is the main factor to lack of testicular self-examination as they were never taught or ever heard about it. Uganda as a developing country has high levels of adolescents who lack knowledge about testicular cancer and TSE. This is in agreement with the study by [18] where most participants (87%) reported a lack of the skill for performing TSE and others perceived TSE as an embarrassing and time consuming procedure. Breast self-examination (BSE) is gaining much attention among women in the sub-Saharan region but knowledge of TSE in men in the same region remains very poor [19].

It was observed that young males who come to the hospitals, some of them have testicular disorders and most of those males don’t know that they have the disorders. It has been shown that young males in Uganda lack knowledge and majority not to be practicing TSE. This is reflected by a study done in 2012 among Ugandan male university students whereby 71% of the sample did not know when to perform TSE and 80% reported to lack the skill for performing TSE and only 14% were performing TSE regularly [18]. Studies have been carried out in higher institutions of learning about TSE but information is lacking for younger males in secondary schools.

If young males have low levels of knowledge, it means that the practice of TSE will be poor and this will consequently lead to late detection of testicular tumors or other scrotal abnormalities. While TSE is often recommended, there is surprisingly little data available concerning TSE knowledge and awareness among male populations. This study therefore sought to assess the level of knowledge and practice of TSE among students at Ntare School in Mbarara District, south western Uganda.

## Methods

### Study population and setting

This study used a descriptive cross-sectional study design. The study employed quantitative methods of data collection among male students in advanced level (A’ level) at Ntare School who met the eligibility criteria for the study. The study was conducted in Ntare School. Ntare School is a public boys' secondary school located in Mbarara District, south western Uganda. The school is approximately 1 kilometre, by road, north of Mbarara District town. The school campus is approximately 267 kilometres, by road, south west of Kampala, Uganda's capital and largest city. The school has both ordinary level (O’ level) and advanced level (A’ level). The average age for O’ level students is 15 years and A’ level is 19 years. Ntare School was selected because it’s the most prominent school in western Uganda thus it has students from all regions of the country. Therefore this study was to be a representative of all regions of Uganda. In addition, it is a single sex (males) school and near the regional referral hospital. Sample size was estimated by Yamane formula (1967:886) based on similar studies.

$$n=\frac{N}{1+N{\left(e\right)}^{2}}$$

Where; n = sample size, N = population size of students in advanced level, e = level of error expected which is 0.05.

$$n=\frac{280}{1+280{\left(0.05\right)}^{2}}$$

For convenience in data collection, a rounded figure of 165 participants was considered.

### Data collection

The data was collected using a self-administered questionnaire to the respondents. Each questionnaire was numbered before giving it to the respondent. Once a respondent accepted and consented to participate in the study, he was given a questionnaire and clarification was made by the researcher to the respondents where needed. The questionnaire had 14 questions and filling the questionnaire lasted about 15 minutes for each respondent. By the end of the day, the filled questionnaires were kept in a safe place and the whole exercise took one month until a required sample size was reached.

### Data management

Data analysis was done using Statistical Package for Social Scientists (SPSS) Version 16.0. Findings of the study were presented in form of tables, graphs and pie charts.

### Quality control

**Reliability of data:** pretesting of questionnaires was done before the actual exercise of data collection started. The study tool was pretested on 10 male selected students at Mbarara High School and the gaps identified were addressed accordingly. The questions were made simple, concise and the respondents were exposed to the questionnaire only once.

**Validity of data:** the questionnaire selected to collect data was designed to contain all the information to cater for all the objectives of the study. The questionnaire was designed with the help of the research supervisor in order to make sure that all the necessary information was included.

### Ethical consideration

Mbarara University of Science and Technology, Faculty Research Ethical Committee (FRC) approved the conduct of the study. Authority from the head teacher of Ntare School was sought to conduct this study in this setting. Informed consent was obtained from respondents before administering the questionnaire to them and all ethical issues were addressed prior.

### Limitations

Since the study targeted only advanced level (A’ level) students, some males who were eligible for the study were left out because they are in ordinary (O’ level) at Ntare School yet they have the same risk of testicular cancer.

## Results

A total of 165 male students in Advanced level (A’ level) at Ntare School were recruited from 15^st^ May 2017 to 15^th^ June 2017. Data collection was done in one month. The respondent rate was 100%. Results are presented in form of tables, graphs and pie charts. The parameters used from demographic characteristics in this study included: age of students, religion, tribe and district. These are presented in the sub sections below:

**Age of the respondents:** out of the total sample of participants, majority; 97.0% (n = 160) were in the age range of 18 - 20 years, 1.8% (n = 3) were in the age range of 21- 23 years and 1.2% (n = 2) were in the age range of 24 - 26 years.

**Figure 1:**
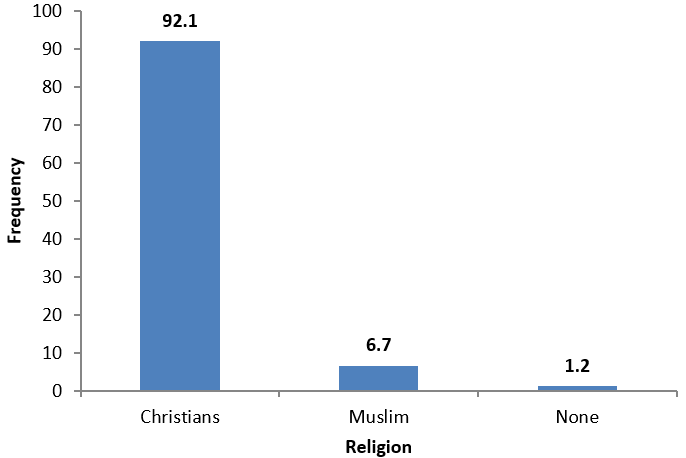
Religions of the respondents

**Religions of the respondents:** as displayed in Figure 1, 152 (92.1%) of the respondents were Christians, 11 (6.7%) were Muslims and 1.2% had no religion.

**Tribes of the respondents:** as seen from Table 1, majority of respondents were Banyankole 102 (61.8%), followed by Bakiga 33 (20.0%), Baganda 10 (6.1%) and other tribes.

**Table 1:** Showing tribe of respondents

	Frequency	Percentage
Muganda	10	6.1 (%)
Mukiga	33	20.0 (%)
Mukonzo	4	2.4 (%)
Munyankole	102	61.8 (%)
Other tribes	16	9.7 (%)
Total	165	100.0 (%)

**Regions where respondents stay during their holidays:** a bigger percentage of the respondents were from south western 50 (30.3%), western 15 (9.1%), central 21 (12.7), eastern 3 (1.8%) and only 1 (0.6%) northern as shown in the Table 2.

**Table 2:** Showing regions of Uganda where respondents spend their holidays

	Frequency	Percentage
South Western	125	75.8 (%)
Western	15	9.1 (%)
Central	21	12.7 (%)
Eastern	3	1.8 (%)
Northern	1	0.6 (%)
Total	165	100.0 (%)

### Knowledge of students regarding testicular self-examination

**Figure 2:**
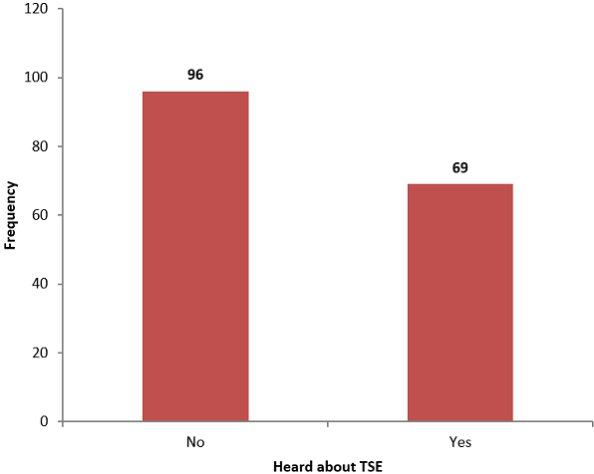
Number of respondents who have ever heard about testicular self-examination (TSE)

In order to track the knowledge on testicular self-examination, respondents were asked different questions and the results were presented in figures. Most of the respondents 96 (58.2%) reported to have never heard about TSE while only 69 (41.8%) reported to have ever heard about TSE as shown in Figure 2.

### Channels through which respondents obtained information about TSE

**Figure 3:**
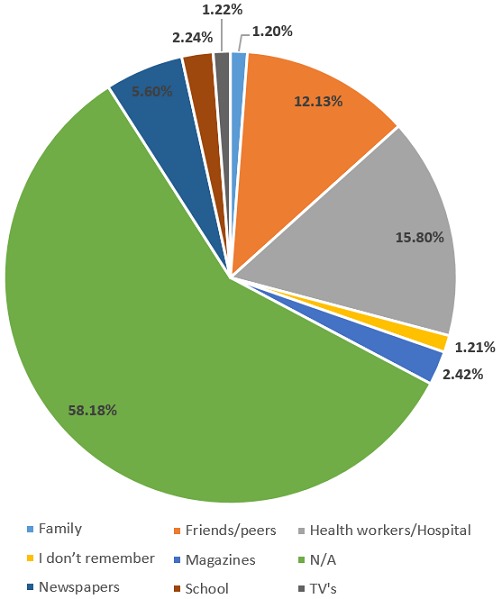
Channels through which respondents obtained information about testicular self-examination (TSE)

Those that had ever heard about TSE, 15.8% (n = 26) had obtained it from health workers/hospital, 12.1% (n = 20) form friends/peers, 5.5% (n = 9) from newspapers, 2.4% (n = 4) form magazines and at school while only 1.2% (n = 2) from their family, TVs and also couldn’t remember as shown in Figure 3.

### Informed a friend about TSE

**Figure 4:**
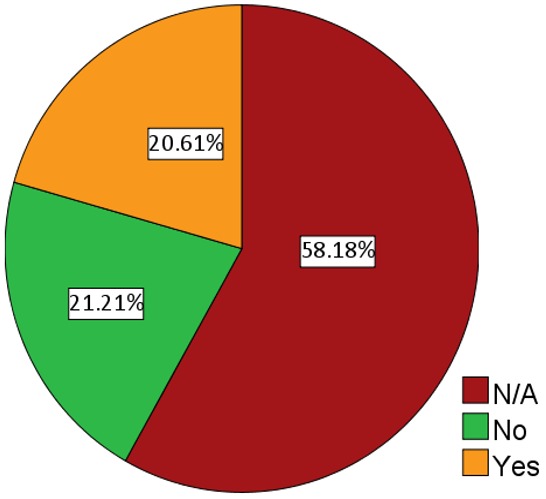
Number of respondents who shared their knowledge about testicular self-examination (TSE) with a friend

Of the 69 respondents who had ever heard about testicular self-examination, 50.7% (n = 35) did not share their knowledge with their friends and 49.3% (n = 34) respondents ever shared their knowledge with others as shown in Figure 4. The most instructions on what to do by respondents given to their friends were; hold the testes and gently press them carefully, followed by to go to the hospital for the test/examination and you just touch the testes and feel whether there is pain.

### Knowledge rating of TSE

**Figure 5:**
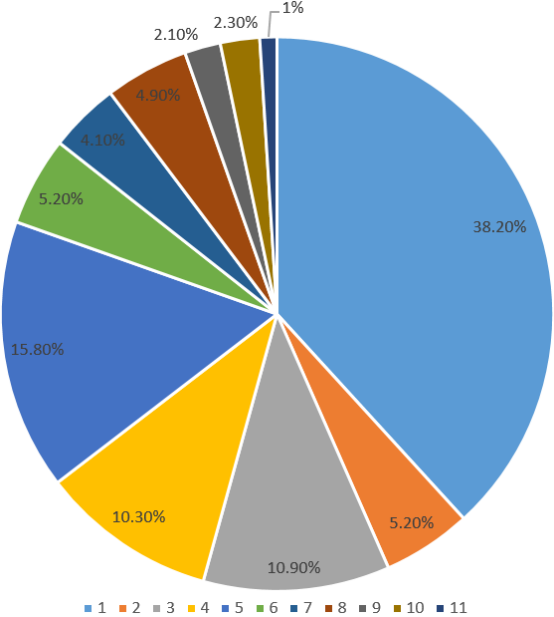
Percentage of knowledge rating about testicular self-examination (TSE) by respondents

A bigger percentage (38.2%) of the respondents rated their knowledge of TSE as 0, followed by 4 as 15.8%, 2 as 10.9%, 3 as 10.3 and others as shown in Figure 5.

### Reasons for respondents that had never heard about testicular self-examination

Of the 96 respondents who reported that they had never heard of TSE, 52 of them simply reported that they didn’t know what it means, 12 said that they lack sensitization about TSE and other reasons as shown in Table 3.

**Table 3:** Showing reasons for respondents that had never heard about testicular self-examination

	Frequency	Percentage
Don’t know what it means	52	31.5 (%)
Have not been having any testicular problem	7	4.2 (%)
I am not well exposed to biological terms	2	1.2 (%)
Lack of sensitization about this topic	12	7.3 (%)
Missing information	21	12.7 (%)
N/A	69	41.8 (%)
Occupied by other stuff like sports	2	1.2 (%)
Total	165	100.0 (%)

### Practice of TSE among students

In order to find out the practice of TSE among students, we asked students to report on their performance of testicular self-examination.

### Performance of testicular self-examination among students

Majority (76.4%) of the respondents reported not to have been performing TSE. Only a small percentage (23.6%) of the respondents reported to be performing TSE.

### Abnormalities that can be found when performing TSE

In the study, respondents were asked to report the abnormalities that could be found when performing TSE. The most reported abnormalities were; any hard particle on the testes 6.7%, swollen testes 4.8%, paining of the testes 4.2%, having one testis 3.0% and others as shown in Table 4.

**Table 4:** Showing abnormalities that can be found when performing testicular self-examination (TSE)

	Frequency	Percentage
Abnormalities like wounds	1	0.6 (%)
Any hard particle on the testis	11	6.7 (%)
Development of whitish skin underneath	1	0.6 (%)
Failure of the testes to withdraw from the body	3	1.8 (%)
Having one testis	5	3.0 (%)
I don't know	14	8.5 (%)
Missing information	111	67.3 (%)
Paining of the testes	7	4.2 (%)
Sometimes testes may not be equal in size	2	1.2 (%)
Swollen testes	10	6.0 (%)
Total	165	100.0 (%)

### How important to perform TSE

Majority 37.0% (61) reported that TSE is very important, 26.1% (43) somewhat important, 17.6% (29) extremely important while 19.4% (32) reported that TSE is not important.

In general, most of the respondents 96 (58.2%) reported lack of knowledge about TSE. Majority of the respondents 72.7% (n =120) reported that they don’t perform TSE at all, followed by a small percentage of 9.7% (n = 16) for those who perform TSE monthly, 8.5% (n = 14) performing TSE annually and 9.1% at least once in 2 years.

## Discussion

This study focused primarily on two objectives: the knowledge and practice of testicular self-examination among students at Ntare School in Mbarara District, south western Uganda. Analysis of respondents by age showed that majority 97.0% were in the age range of 18 - 20 years. This finding was so because most students in advanced level (A’ level) are in that age range as indicated by Ntare School administration that the average age of students in A’ level is 19 years. Banyankole were the majority tribe found in the study and this could simply be due to the fact that they are the predominant ethnic group in the region. There is no significant effect of religion on TSE but religious gatherings could provide a platform for mass education about health promotion programs, TSE inclusive.

Knowledge is an important attribute in health promotion. The efficacy of any health strategy largely relies on how the target population is aware of the strategy and the benefits it can provide. The findings of this study indicate that regarding knowledge about TSE, secondary school male students are less informed as only 41.8% of them have ever heard about TSE.

A search of the literature does not reveal any Ugandan study on secondary school boys for comparison. This low knowledge of TSE may be explained by lack of education to adolescent males that testicular cancer can occur in them and that TSE is a potential screening tool for early detection of testicular cancer and hence, treatment and cure. The results of this study are in line with a study among adults (aged 18 to 50 years old) in three tertiary institutions in Port Harcourt (Nigeria) on 750 males that revealed a similar low knowledge levels (awareness) of TSE concerning the respondents [17]. In that study, only 69 (9.2%) of the respondents were aware that testicular lump could be a sign of TC. The finding of low knowledge with regard to TSE is not surprising, given that a similar low level of awareness has been reported in several studies conducted in developed countries with highly literate populations. For instance, a comparative study of British and Zimbabwean undergraduates revealed that knowledge of TSE was low in both groups of respondents [20].

Another study in Turkey revealed poor knowledge of TSE among college male adolescents [6]. This could be attributed to the fact that the health workers themselves do not instruct their male patients about TSE and encourage them to undertake regular monthly testicular examination as shown in this study whereby only 15.8% of the respondents had received information about TSE from health workers. This view is reinforced by the report of a study in which only 17.5% of physicians surveyed taught TSE to adolescent male patients on a routine basis [21]. In that study, 82.0% of the physicians stated that they were not familiar with the technique of TSE. Health workers also need not only use the hospital health education talks to deliver knowledge to students but also utilize communication channels like radios that are accessed by most of the respondents in our local setting. This is due to the reason that no respondents were found to have received information about TSE via this media under the data analysis.

However the findings of this study are not in agreement with the findings of a study done by [1] among male medical students of the University of Nigeria showed that majority 110 (64%) of the respondents had good knowledge level of testicular self-examination but this could be explained by the fact that the study was carried out among medical students. In this study, nearly more than three quarters of the respondents (80.0%) rated their knowledge of TSE to be below 5 (from 1-10) indicating a low awareness about the subject. The implication of the low rating of the knowledge of TSE is attributable to the lack of health education on TSE provided by health care workers as it was found out by the number of students who had never heard about TSE. However educating the students on the TSE has a high potential to succeed as an important health promotion tool. As found in previous studies by [18], overall, male students gave an average knowledge rating (5.6, from 1-10) of TSE. Knowledge of TSE would largely bridge the gap of global testicular cancer inequality, given that the most important tool for bridging this gap is initiating early detection of TC among the public through TSE.

### Practice of TSE among students

Objective two of the study was to determine the practice of testicular self-examination among students at Ntare School in Mbarara District, Uganda. The findings of the study revealed that 39 (23.6%) of the students perform testicular self-examination. This shows a low practice of TSE among the students. Poor practice of TSE observed among the respondents is as a result of low knowledge of TSE. Examining one’s testicles through physical examination as well as regular screening is recommended for all men over the age of 15 to 50 years. Examination of the testicles on a regular basis by health care provider or by self is the safest and most effective route of prevention. The findings of this study is in line with a study conducted by [14] on 165 Hispanic college students to assess the prevalence of TSE. The study revealed that 36% of the respondents reported that they performed TSE while 64% reported never to be performing TSE. The finding is also in agreement with the work of [22] in Europe on Dutch young males aged 15-19 years. The results show that only 2% of the respondents reported performing TSE. After hearing about TSE through the questionnaire, 41% of respondents reported to have a positive intension to start performing TSE. The study is also in agreement with the study by [3]. The result stated that 46% of respondents reported performing TSE and 51% reported not performing TSE.

The results of this study indicate that 61 (37.0%) of the respondents stated that TSE is very important to perform showing that with appropriate knowledge, then students would be willing to perform TSE monthly as recommended. The reasons affecting the practice of TSE are attributable to lack of skill for performing TSE. The findings of this study is in line with the study by [18] in Uganda on 323 male students in universities of Uganda. Most participants (87%) reported a lack of skill for performing TSE, 80% perceived TSE as embarrassing and 79% perceived TSE as time consuming. The poor performance of TSE can be attributed to poor/lack of health education, workshops, seminar or conferences where people/students will be taught on the importance and practice of TSE of which absence of these impede the awareness and practice of TSE.

Results of the current study indicate that majority of the respondents 120 (72.7%) reported that they don’t perform TSE at all, followed by a small number of 16 (9.7%) for those who perform TSE monthly, 14 (8.5%) performing TSE annually, 4.2% (7), 5 (3.0%) perform TSE every 6 months and 3 (1.8%). A small percentage 9.7% indicates students who perform TSE monthly which is bigger than all those other students that perform TSE as recommended, to be performed monthly. The findings of this study are in line with a study carried out among a sample of male university students across 5 low, middle and high income countries. The study found low levels of TSE practice, with 13.6% practicing TSE in the past 12 months, which compares with most previous surveys among male university students [9, 18]. In addition, the results of this study indicate students’ report about testicular abnormalities that could be found in the process of performing TSE for example swollen testes, any hard particle on the testis, painful testes and undescended testis.

### Recommendation

Based on the findings in the study, the authors of this study suggested that policy makers in Uganda need to package health education sessions in secondary school curriculum about testicular self-examination. Teachers also need to organize seminars and conferences to enlighten students on the importance and practice of TSE. There should be encouragement and health education by health workers who are familiar with the proper technique of performing TSE. Such teaching could be incorporated into routine outpatient and inpatient interactions with high risk clients. Public health campaigns should encourage more men to perform regular TSE. In addition, young men attending healthcare institutions for any reason should be given health education on TSE, perhaps accompanied by a patient leaflet.

## Conclusion

In this study, the knowledge and practice of TSE were low among adolescent secondary school boys in Ntare School, south western Uganda suggesting that these students are unaware of the value of this personal health promotion tool. Knowledge about testicular self-examination should be increased for younger men through providing information to them and if done, young males would be willing to participate in taking preventive measures.

### What is known about this topic

- It has been shown that young males in Uganda lack knowledge and majority do not practice Testicular self-examination. This is reflected by a study done in 2012 among Ugandan male university students whereby 71% of the sample did not know when to perform TSE and 80% reported to lack the skill for performing TSE and only 14% were performing TSE regularly;
- Studies have been carried out in higher institutions of learning about TSE but information is lacking for younger males in secondary schools in Uganda.

### What this study adds

- If young males have low levels of knowledge, it means that the practice of TSE will be poor and this will consequently lead to late detection of testicular tumors or other scrotal abnormalities such as varicoceles, hydroceles and swellings;
- Therefore the findings of this study will act as an eye opener to the young people to help in early diagnosis of testicular cancer plus other scrotal abnormalities and contribute to the sensitization of males about TSE in Ugandan secondary schools.

## Competing interests

The authors declare no competing interests.
